# High-accuracy deep ANN-to-SNN conversion using quantization-aware training framework and calcium-gated bipolar leaky integrate and fire neuron

**DOI:** 10.3389/fnins.2023.1141701

**Published:** 2023-03-08

**Authors:** Haoran Gao, Junxian He, Haibing Wang, Tengxiao Wang, Zhengqing Zhong, Jianyi Yu, Ying Wang, Min Tian, Cong Shi

**Affiliations:** ^1^The School of Microelectronics and Communication Engineering, Chongqing University, Chongqing, China; ^2^State Key Laboratory of Computer Architecture, Institute of Computing Technology, Chinese Academy of Sciences, Beijing, China

**Keywords:** neuromorphic computing, spiking neural network, ANN-to-SNN conversion, deep SNNs, quantization-aware training

## Abstract

Spiking neural networks (SNNs) have attracted intensive attention due to the efficient event-driven computing paradigm. Among SNN training methods, the ANN-to-SNN conversion is usually regarded to achieve state-of-the-art recognition accuracies. However, many existing ANN-to-SNN techniques impose lengthy post-conversion steps like threshold balancing and weight renormalization, to compensate for the inherent behavioral discrepancy between artificial and spiking neurons. In addition, they require a long temporal window to encode and process as many spikes as possible to better approximate the real-valued ANN neurons, leading to a high inference latency. To overcome these challenges, we propose a calcium-gated bipolar leaky integrate and fire (Ca-LIF) spiking neuron model to better approximate the functions of the ReLU neurons widely adopted in ANNs. We also propose a quantization-aware training (QAT)-based framework leveraging an off-the-shelf QAT toolkit for easy ANN-to-SNN conversion, which directly exports the learned ANN weights to SNNs requiring no post-conversion processing. We benchmarked our method on typical deep network structures with varying time-step lengths from 8 to 128. Compared to other research, our converted SNNs reported competitively high-accuracy performance, while enjoying relatively short inference time steps.

## 1. Introduction

Deep learning technology has achieved unprecedented success in versatile intelligent applications in modern society. However, the real-valued deep artificial neural network (ANN) models are quite power-hungry due to their intensive matrix multiplication operations (LeCun et al., 2015). By contrast, neuromorphic computing with spiking neural networks (SNNs) is more promising for ubiquitous cost- and energy-constrained mobile, embedded, and edge platforms (Roy et al., 2019). The SNN adopts spatiotemporally sparse spike events to encode, transmit, and process information the way human brain cortical neurons do.

However, training deep SNNs is highly challenging because it is difficult to directly apply the backpropagation (BP) method to SNNs owing to the inherent discontinuity of discrete spikes. A common indirect approach to overcome this problem is to train a structurally equivalent ANN model offline and then convert it to an SNN with the learned synaptic weights for inference, where the real values of inputs and outputs of ANN neurons correspond to the rates of presynaptic (input) and postsynaptic (output) spikes of the SNN neurons (Diehl et al., 2015; Hunsberger and Eliasmith, 2016; Rueckauer et al., 2017; Zhang et al., 2019; Han and Roy, 2020; Han et al., 2020; Kim et al., 2020; Lee et al., 2020; Yang et al., 2020; Deng and Gu, 2021; Dubhir et al., 2021; Ho and Chang, 2021; Hu et al., 2021; Kundu et al., 2021; Li et al., 2021b; Bu et al., 2022; Liu et al., 2022). Although previous ANN-to-SNN techniques usually obtain state-of-the-art object recognition accuracies, they require complicated post-conversion fixations such as threshold balancing (Diehl et al., 2015; Rueckauer et al., 2017; Han et al., 2020; Liu et al., 2022), weight normalization (Diehl et al., 2015; Rueckauer et al., 2017; Ho and Chang, 2021), spike-norm (Sengupta et al., 2019), and channel-wise normalization (Kim et al., 2020), to compensate the behavioral discrepancies between artificial and spiking neurons. In addition, a few of those methods require a relatively long time window (e.g)., 2,500 algorithmic discrete time steps (Sengupta et al., 2019), allowing for sufficient spike emissions to precisely represent the real values of the equivalent ANNs. This incurs high latencies and additional computational overheads, severely compromising the efficiency of SNNs.

To mitigate the aforementioned overheads in ANN-to-SNN conversion, this study proposes a simple and effective deep ANN-to-SNN framework without any post-conversion tuning, and the converted SNN can achieve a high recognition accuracy in a relatively shorter temporal window (i.e., 128 down to 8 time steps). This framework adopts our proposed calcium-gated bipolar leaky integrate and fire (Ca-LIF) spiking neuron model to well approximate the function of the ReLU neuron widely used in deep ANNs. It fully leverages off-the-shelf quantization-aware training (QAT) toolkit to train the ANNs with low-bit precision ReLU activations, which can be captured as the spike rate of the Ca-LIF neuron in an intermediately short time window.

The rest of this article is organized as follows: Section 2 explains the background of neural networks, including the ReLU and the basic LIF neurons. Section 3 proposes our Ca-LIF spiking neuron model and the QAT-based ANN-to-SNN framework, which are validated with elaborate experiments mentioned in Section 4. Section 5 summarizes this study.

## 2. Preliminaries

### 2.1. Convolution neural network

**Figure 1:**
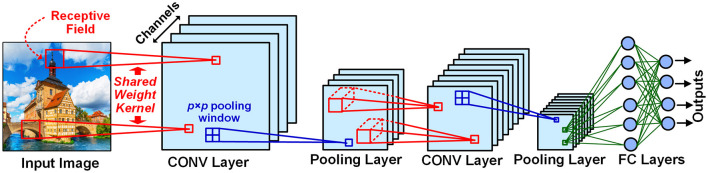
Typical structure of deep neural networks. In convolutional (CONV) layers, the Receptive Field (RF) is a spatial neighborhood around a neuron in a channel connected by a shared weight kernel to the next layer's neurons. The pooling layer is utilized to reduce the size of its preceding CONV layer feature map by a pooling window. Each neuron in a fully connected (FC) layer are connected to all the neurons in its previous layer. The outputs of the final layer indicate the image object recognition result.

The typical structure of a deep neural network (shown in Figure 1) is composed of alternating convolutional (CONV) layers for feature detection and pooling layers for dimensionality reduction, followed by stacked fully connected (FC) layers as a feature classifier. In a CONV layer, each neuron in a channel is connected *via* a shared weight kernel to a few neurons within a spatial neighborhood called receptive field (RF) in the channels of the precedent layer. In a pooling layer, each neuron aggregates the outputs of the neurons in a *p* × *p* spatial window from the corresponding channel of its precedent CONV layer, thereby realizing data dimensionality reduction and small translational invariance. In an FC layer, each neuron is fully connected to all neurons in its precedent layer. The neuron with the most active outputs in the final layer indicates the recognition result.

### 2.2. ReLU neuron in ANN

**Figure 2:**
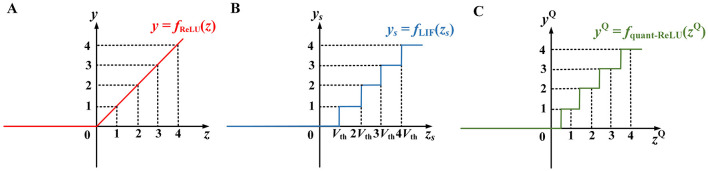
Input–output relationships of **(A)** the ReLU neuron, **(B)** the basic LIF neuron, and **(C)** the quantized ReLU approximation based on rounding (Deng and Gu, 2021). In **(A)**, when the input *z* > 0, the output *y* = *z*, otherwise *y* = 0. In **(B)**, *z*_S_ is the integrated membrane across the total time steps and *V*_th_ is the threshold. In **(C)**, *z*^Q^, *y*^Q^ are the input and output of the quantized ReLU function based on rounding.

The output of the ReLU neuron widely used in ANNs is formulated as follows:


(1a) $$\begin{array}{l} y=f_{\text{ReLU}}\left(z\right)=max\left(0,z\right) \end{array}$$

where *z* is the *net summation* which is calculated as follows:


(1b) $$\begin{array}{l} z=\underset{i}{\sum }w_{i}x_{i}+b \end{array}$$

with *x*_*i*_ as the *i*-th input value to the neuron, *w*_*i*_ the connecting weight, and *b* a bias term. Figure 2A depicts the ReLU function.

### 2.3. Basic LIF neuron in SNN

The LIF neuron is the most commonly adopted model in SNNs (Roy et al., 2019). It is biologically plausible with an internal state variable called membrane potential *V*_m_ (initialized to 0 at the beginning of spike trains of every input image) and exhibits rich temporal dynamics. Once the neuron receives a spike event *via* any of its synapses, the corresponding synaptic weight *w*_*i*_ is integrated into its *V*_m_. Meanwhile, the neuron linearly leaks all the time. The event-driven LIF model can be described as follows:


(2) $$\begin{array}{l} V_{\text{m}}\left(t_{k}\right)=V_{\text{m}}\left(t_{k-1}\right)+w_{i\left(k\right)}-\lambda \left(t_{k}-t_{k-1}\right) \end{array}$$

where *t*_*k*_ and *i*(*k*) are the algorithmic discrete time step and the index of the synapse when and where the *k*-th input presynaptic spike arrives, respectively, and λ is a constant leakage at every time step. Whenever *V*_m_ crosses a pre-defined threshold *V*_th_ > 0, the neuron fires a postsynaptic spike to its downstream neurons and resets *V*_m_ by subtracting *V*_th_ from it. Suppose an input image has a presentation window of *T* time steps (i.e., the length of spike trains encoded from the image pixels), one would estimate the total output spike count of the LIF neuron as follows (Han et al., 2020; Lee et al., 2020):


(3a) $$\begin{array}{l} y_{\text{S}}=f_{\text{LIF}}\left(z_{\text{S}}\right)=\max\left(0,\text{floor}\left(z_{\text{S}}/V_{\text{th}}\right)\right) \end{array}$$

where *floor* returns the largest integer no larger than its argument, and *z*_s_ is the *net integration* across all the *T* time steps:


(3b) $$\begin{array}{l} z_{\text{S}}=\underset{k}{\sum }w_{i\left(k\right)}-\lambda T=\underset{i}{\sum }w_{i}x_{\text{S}i}-\lambda T \end{array}$$

with *x*_si_ being the total count of input spikes *via* synapse *i*. Equation (3a) is depicted in Figure 2B.

## 3. Materials and methods

### 3.1. Motivation

From the similarities between Eqs. (1) and (3) and between Figures 2A, B, it appears that the LIF neuron can be used to approximate the ReLU function by treating its pre- and postsynaptic spike rates or counts *x*_si_, *y*_s_ as ReLU's input and output values *x*_*i*_, *y*. The leakage term -λ*T* in Equation (3b) acts as the bias *b* in Equation (1b). Thus, we can first train a deep ANN using standard BP, and then export the learned weights and biases to a structurally equivalent SNN of LIF neurons for inference.

However, there are three challenges hindering such a direct ANN-to-SNN conversion:

**1)** The input and output spike counts of the LIF neuron are discrete integers, while ReLU allows continuous-valued inputs and output. Particularly, the *y*_s_ in Figure 2B is a scaled (by the factor of 1/*V*_th_) and staircase-like approximation of the ReLU output *y* in Figure 2A. To reduce their discrepancy, a long time window is often needed to generate sufficient output spikes, resulting in a high inference latency.

**Figure 3:**
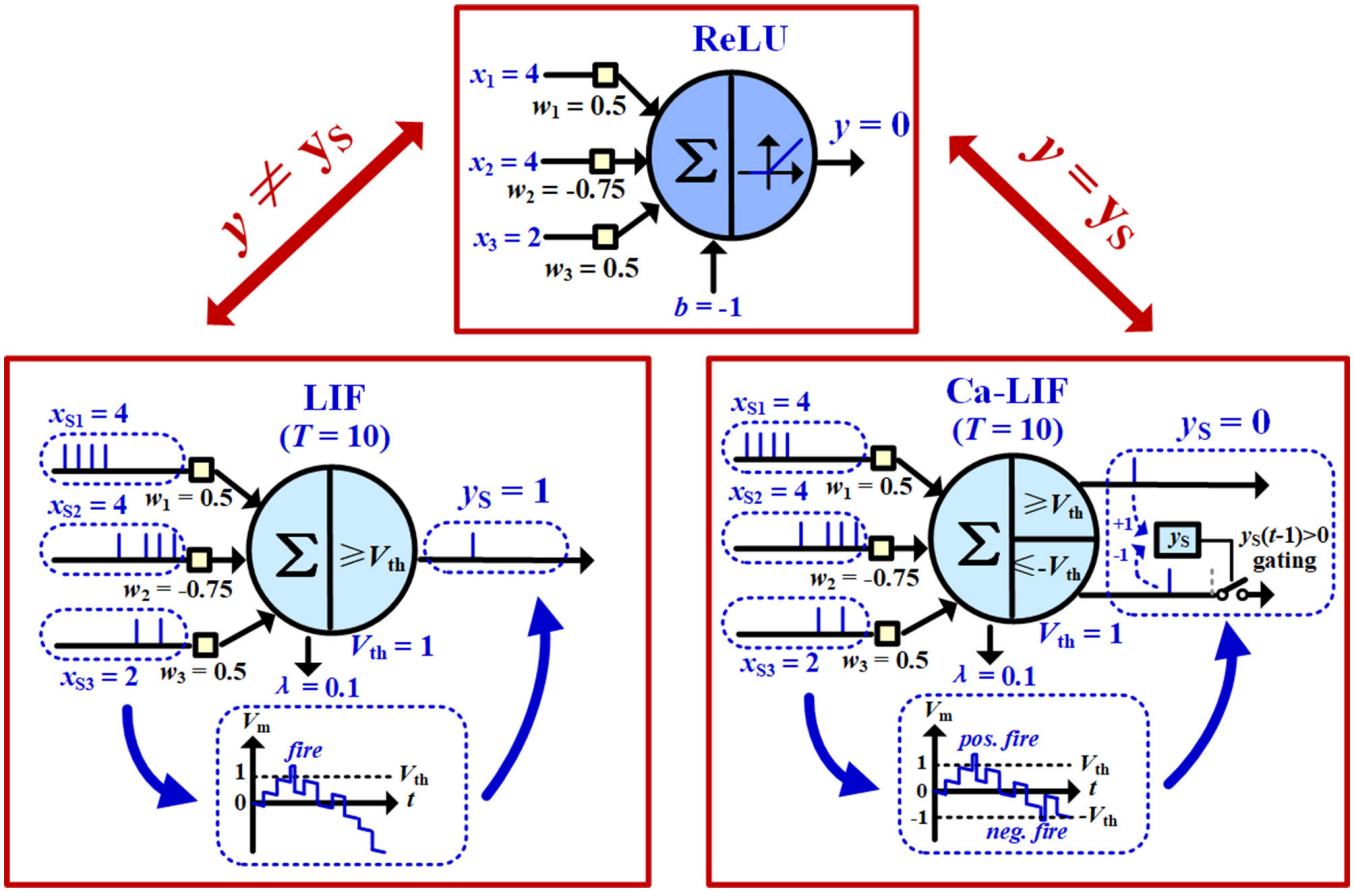
Comparison of the input–output relationships of the ReLU neuron, the basic LIF neuron, and the proposed Ca-LIF neuron.

**2)** Due to the extra temporal dimension of the LIF neuron, Equation (3a) may be significantly violated sometimes. As illustrated in Figure 3, the earlier input spikes *via* positive synaptic weights trigger output spikes, which could not be canceled out by later input spikes *via* more negative synaptic weights, as the information accumulated into the negative *V*_m_ of the LIF neuron cannot be passed on to other neurons *via* any output spikes. Therefore, even when the LIF neuron has weights and inputs values *x*_si_ = *x*_*i*_ identical to those of the ReLU neuron, with a leakage constant set to be λ = -*b*/*T*, LIF output spike count *y*_s_ still severely deviates from ReLU output *y* and largely violates Equation (3a).

**3)** Note that there is a floor(*z*_s_/*V*_th_) operation in Equation (3a) due to the discrete fire thresholding mechanism, leading to a shift of *V*_th_/2 along the positive *z*_s_ axis in Figure 2B, compared to the rounding-based quantized ReLU approximation as shown in Figure 2C (Deng and Gu, 2021). Indeed, a better approximation to the ReLU neuron expects a round operation instead of the floor function to obtain statistically zero-mean quantization errors (Deng and Gu, 2021).

To overcome the first challenge, we can leverage QAT ANN training toolkits to produce an ANN with low-precision ReLU outputs, while minimizing the accuracy loss compared to a full-precision ANN. The complete QAT-based ANN-to-SNN framework is proposed in Section 3.3. For the other two challenges, we propose a Ca-LIF neuron model. It reserves the spike-based event-driven nature of a biological neuron, while mathematically better aligning with the (quantized) ReLU curve regardless of the input spike arrival order, as introduced later.

### 3.2. The proposed Ca-LIF spiking neuron model

We proposed the Ca-LIF spiking neuron model to correct the output mismatches between the basic LIF model and the quantized ReLU function, as exhibited in Figure 3. It performs the same leaking and integration operations as in Equation (2) but employs a slightly different firing mechanism. The Ca-LIF neuron holds symmetric thresholds *V*_th_ > 0 and -*V*_th_ < 0. Once its *V*_m_ up-crosses *V*_th_, or down-crosses -*V*_th_ with the gating condition *y*_S_(*t*-1) > 0 satisfied, the neuron fires a *positive* or *negative* spike, respectively, where *y*_S_ in the Ca-LIF neuron represents *signed* output of the spike count. i.e., the positive output spike count minus the positive negative spike count. Actually, *y*_s_ resembles the *calcium ion concentration* (Ca^+^) in a biological neuron (Brader et al., 2007). Note that if a Ca-LIF neuron receives a negative spike sent by another neuron *via* its synapse *i*, -*w*_*i*_ is instead integrated onto the *V*_m_ in Equation (2). This neuron resets by adding *V*_th_ to the *V*_m_ after it fires a negative output spike.

Moreover, as mentioned earlier, the spiking neuron should perform a rounding function to replace the floor operation on (*z*_s_/*V*_th_) in Equation (3a) to better align with the quantized ReLU behavior. Mathematically, the Ca-LIF neuron should execute:


(3c) $$\begin{array}{l} y_{\text{S}}=f_{\text{LIF}}\left(z_{\text{S}}\right)=\max\left(0,\text{round}\left(z_{\text{S}}/V_{\text{th}}\right)\right) \end{array}$$

To achieve this, after all the spike events input to the SNN composed of Ca-LIF neurons have been processed, each Ca-LIF neuron in the first SNN layer with their *V*_m_ between *V*_th_/2 ~ *V*_th_ (or between -*V*_th_ ~ -*V*_th_/2, and *y*_S_ > 0) is a force to fire a positive (or negative) spike. These *rounding* spikes propagate to other Ca-LIF neurons in subsequent layers, trying to trigger their own rounding spikes based on their halved thresholds ±*V*_th_/2. This progresses until the final layer is completed.

### 3.3. QAT-based ANN-to-SNN conversion framework

Using the aforementioned Ca-LIF neurons, we now propose the details of the simple QAT-based ANN-to-SNN conversion framework. First, utilize any off-the-shelf QAT toolkit available to train a deep quantized ANN. Next, export the learned ANN weights to an SNN composed of Ca-LIF neurons organized in the same network structure as the ANN, and analytically determine the neuron thresholds. Typically, a QAT toolkit would provide the low-bit precision mantissa $w_{i}^{Q}$ associated with a scaling factor *S*_*w*_ of each learned quantized weight in the ANN, as well as the bias *b*, the input and output scaling factors *S*_*x*_ and *S*_*y*_ of the neurons. One quantized ReLU neuron performs inference with its such learned parameters as follows:


(4) $$\begin{array}{l} \begin{array}{c} y^{\text{Q}}=\max\left(0,\text{round}\left(\underset{i}{\sum }\frac{\left(S_{w}w_{i}^{\text{Q}}\right)\left(S_{x}x_{i}^{\text{Q}}\right)+b}{S_{y}}\right)\right)\\ \;=\max\left(0,\text{round}\left(\underset{i}{\sum }\frac{w_{i}^{\text{Q}}x_{i}^{\text{Q}}+b/S_{w}S_{x}}{S_{y}/S_{w}S_{x}}\right)\right) \end{array} \end{array}$$

Wherein the superscript Q denotes *quantized*. By comparing the forms of Equations (3c) and (4), it can be found that, if we simply set


(5) $$\begin{array}{l} V_{\text{th}}=S_{y}/\left(S_{w}S_{x}\right),\;\lambda =-b/\left(S_{w}S_{x}T\right) \end{array}$$

for a Ca-LIF neuron, it can seamlessly replace the quantized ReLU neuron and reproduce its input–output relationship of Figure 2C in the form of spike counts, with exactly the same learned weights.

In addition, one neuron in an average pooling layer of the quantized ANN performs a quantized linear operation as follows:


(6) $$\begin{array}{l} y_{\text{P}}=\text{round(}\underset{j\in \text{PW}}{\sum }y_{{}_{j}}^{\text{Q}}/p^{2}\text{)}\left(6\right) \end{array}$$

where PW denotes the set of ReLU neurons in the *p* × *p* pooling window connecting to the pooling neuron. Such pooling neuron can also be approximated by our Ca-LIF neuron but without the *y*_s_ gating constraint on negative firing, and with its *V*_th_ being *p*^2^, the leakage constant λ being 0, and all synaptic weights being 1.

## 4. Experiments

### 4.1. Benchmark datasets

We evaluated our method on five image datasets: MNIST, CIFAR-10, CIFAR-100, Caltech-101, and Tiny-ImageNet. Their image resolution, number of object categories, as well as the training/testing subsets partition are listed in Table 1. The MNIST dataset contains 28 × 28 handwritten digit images of 10 classes, i.e., 0–9. It is divided into 50,000 training samples and 10,000 testing samples. The CIFAR-10 dataset contains 10 object classes, including 50,000 training images and 10,000 testing images with an image size of 32 × 32. For CIFAR-100, it holds 100 object classes, each owning 500 training samples and 100 testing samples. The Caltech-101 dataset consists of 101 object categories, each of which holds 40–800 image samples with a size of 300 × 200 pixels. The Tiny-ImageNet benchmark is composed of as many as 200 object classes, each of which has 500 training samples and 50 testing samples with an image size of 64 × 64.

**Table 1:** Benchmark datasets.

**Dataset**	**Pixel resolution**	**^#^ of categories**	**Training samples**	**Testing samples**
MNIST	28 × 28	10	60,000	10,000
CIFAR-10	32 × 32	10	50,000	10,000
CIFAR-100	32 × 32	100	50,000	10,000
Caltech-101^*^	128 × 128	101	6162	1695
Tiny-ImageNet	64 × 64	200	100,000	10,000

* Resized from original 300 × 200 resolution. A Difference-of-Gaussian (DoG) filter is applied to each of the red, green, and blue channels, before the pixels are encoded into spike trains.

# Number of categories.

We employed the inter-spike interval (ISI) coding method (Guo et al., 2021) to encode pixel values into spikes. The pixel brightness *Pix* (for color images, this refers to the color component in each of the red, green, and blue channels) was converted to a spike train with *N* spikes in a *T* time-step window, with *N* = *floor*(α · *T*· *Pix* / *Pix_max*), where function *floor*(*x*) returned the biggest integer no larger than *x, Pix_max* was the maximum value a pixel could reach (for 8-bit image pixels which used in our work, *Pix_max* = 255), and α ≤ 1 controlled the spike rate, which was set to 1 throughout our experiments unless otherwise stated. The *n*-th spike happened at time step *t*_*n*_ = *floor*(*n* · *t*_int_), where *t*_int_ = *T* / (α· *T*· *Pix* / *Pix_max*) = *Pix_max* / (α·*Pix*) ≥ 1 was the temporal interval (non-rounded) between two successive spikes. In particular, the brightest pixel value of 255 would be converted to a spike train of totally *N* = *floor*(α· *T* · 255/255) = *T* spikes with *t*_int_ = α = 1. In other words, its converted spike train reached the maximum rate of one spike per time step.

### 4.2. Network structure configuration

We adopted five typical deep network structures to evaluate our Ca-LIF spiking neuron and ANN-to-SNN framework: (1) *Lenet-5* (Lecun et al., 1998), (2) *VGG-9* (Lee et al., 2020), (3) *ResNet-11H*, which only kept half of the channels in each CONV layer of the Resnet-11 (Lee et al., 2020), and (4) *MobileNet-20*, a reduced version of MobileNetV1 (Howard et al., 2017) with the original 16th – 23rd CONV layers removed, and (5) *VGG-16*. We modified all pooling layers in these networks to perform average pooling. Moreover, for each network, the kernel size in its first layer and the number of neurons in its last FC layer had to accommodate the image size (i.e., the image resolution and number of color channels) and the number of object categories, respectively, when coping with different image datasets.

### 4.3. Recognition accuracy

In our experiments, we leveraged the off-the-shelf PyTorch QAT toolkit (PyTorch Foundation, 2022) to train deep ANNs of the aforementioned five neural network structures, and then exported the learned parameters to construct structurally equivalent SNNs for inference. The learned weights were directly translated to the synaptic weights of SNN Ca-LIF neurons, while other parameters like the biases and quantization scaling factors were used to determine the thresholds and leakages of Ca-LIF neurons according to Equation (5).

**Figure 4:**
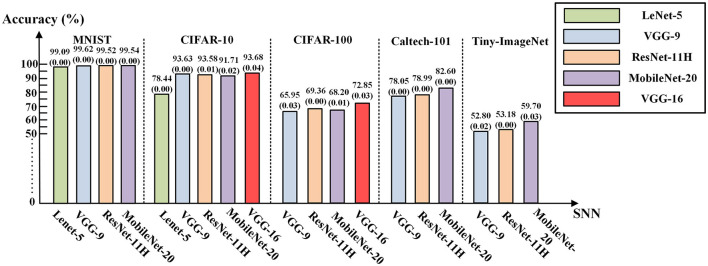
The testing accuracies of the SNNs (*T* = 128) converted from quantized ANNs. The numeric in the bracket below the accuracy is the loss of the converted SNN compared with the corresponding quantized ANN.

The PyTorch QAT toolkit quantized the inputs, outputs, and weights of the ANN neurons all into a signed 8-bit format during training. Note that we can freely leverage any other available QAT toolkit supporting other ANN activation bit-precisions, including binary and ternary activations. We employed the standard stochastic gradient descent method to train ANNs with a momentum of 0.9. The batch normalization (BN) (Ioffe and Szegedy, 2015) technique was also employed in the QAT training to improve the accuracy performance of some deep networks on complex datasets. The BN layers' parameters were updated with other parameters in a unified QAT process and were already incorporated into the convolution layers' biases and quantized 8-bit weights before being exported to SNNs. The training of the QAT starts from scratch rather than relying on transferring learning. For converted SNN inference, we set *T* = 128 time steps as the baseline spike encoding window length. The testing accuracies of the SNN (*T* = 128) under each network structure configuration in Section 4.2 are demonstrated in Figure 4. These results indicated that our ANN-to-SNN conversion framework along with the proposed Ca-LIF neuron model achieved competitively high recognition performance. Indeed, the accuracy gap of the converted SNNs (*T* = 128) and their pre-conversion quantized ANN counterparts was negligibly below 0.04%. The results of experiments on MNIST, CIFAR-10, CIFAR-100, Caltech-101, and Tiny-ImageNet demonstrate the superiority and universality of our method.

**Figure 5:**
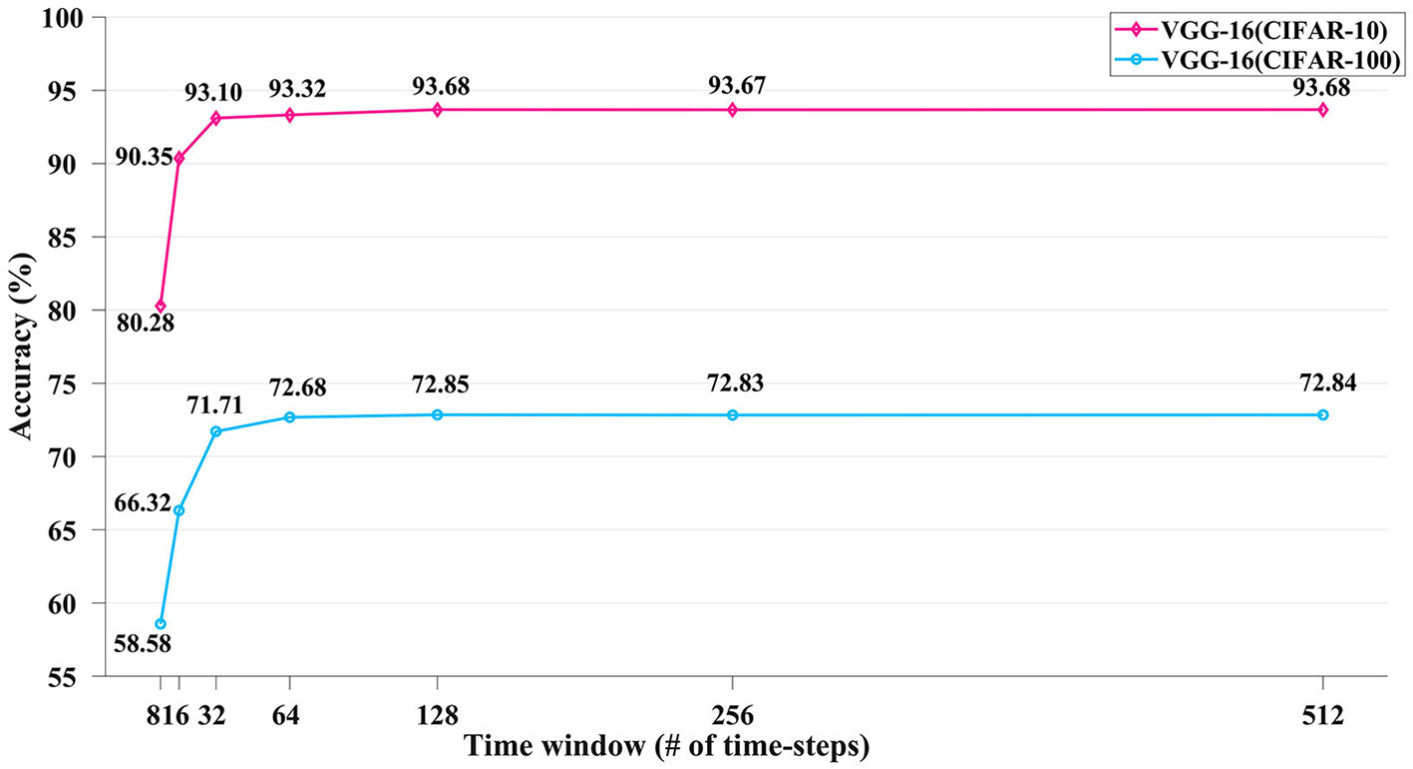
The inference accuracy performance of the converted VGG-16 SNN on the CIFAR-10 and CIFAR-100 datasets using varying numbers of time-steps.

Moreover, to evaluate the accuracy vs. latency (i.e., the number of inference time steps) tradeoff of our converted SNNs, Figure 5 depicts the accuracies of our converted VGG-16 SNN on the CIFAR-10 and CIFAR-100 image datasets under different time window length configurations with varying time steps of *T* = 8 to 512. The accuracies saturate above *T* = 128, as we utilized a signed 8-bit activation for the pre-conversion quantized ANN. A more elaborate work comparison and discussion about this is provided in Section 4.4.

### 4.4. Work comparison and discussion

Table 2 compares our work with other previous ANN-to-SNN conversion research. Since a quantized ANN itself may suffer a bit lower accuracy (sometimes a little higher) than its full-precision version, we also trained and tested the recognition accuracies of full-precision ANNs using the aforementioned network structures for a fair comparison, and further evaluated the accuracy loss between the converted SNNs and corresponding full-precision ANNs.

**Table 2:** Accuracy comparison between our and other ANN-to-SNN conversion methods.

**Dataset**	**Ref**.	**Network Structure**	**Spiking Neuron Model**	**# of Time-Steps**	**SNN Acc**.	**Full-precision ANN Acc**.	**Acc. Loss ANN% -SNN%)**
MNIST	Diehl et al. (2015)	LeNet-5	IF^*^	500	99.12%	99.14%	0.02%
	Sengupta et al. (2019)	LeNet-5	IF^*^	2500	99.59%	99.57%	−0.02%
	Hu et al. (2021)	ResNet-8	IF^**^	350	99.59%	99.59%	0.00%
	**Ours**	**LeNet-5**	**Ca-LIF** ^**^	**128**	**99.09%**	**99.07%**	**-0.02%**
	**Ours**	**VGG-9**	**Ca-LIF** ^**^	**128**	**99.62%**	**99.59%**	**-0.03%**
	**Ours**	**ResNet-11H**	**Ca-LIF** ^**^	**128**	**99.52%**	**99.48%**	**-0.04%**
	**Ours**	**MobileNet-20**	**Ca-LIF** ^**^	**128**	**99.54%**	**99.48%**	**-0.06%**
CIFAR-10	Diehl et al. (2015)	ResNet-11	IF^*^	500	90.98%	91.87%	0.82%
	Sengupta et al. (2019)	ResNet-11	IF^*^	2500	91.65%	91.87%	0.22%
	Kundu et al. (2021)	ResNet-12	LIF^**^	100	90.79%	92.04%	1.25%
	Diehl et al. (2015)	VGG-9	IF^*^	500	91.89%	91.98%	0.09%
	Sengupta et al. (2019)	VGG-9	IF^*^	2500	92.01%	91.98%	−0.03%
	Kundu et al. (2021)	VGG-11	LIF^**^	100	89.84%	91.57%	1.73%
	Sengupta et al. (2019)	ResNet-20	IF^*^	2500	87.46%	89.10%	1.64%
	Han et al. (2020)	ResNet-20	IF^**^	2048	91.36%	91.47%	0.11%
	Bu et al. (2022)	ResNet-20	IF^**^	64	92.35%	N/A	N/A
	Deng and Gu, 2021)	ResNet-20	IF^**^	128	93.56%	92.31%	−1.25%
	Li et al. (2021a)	ResNet-20	IF^**^	128	95.42%	95.46%	0.04%
	Sengupta et al. (2019)	VGG-16	IF^*^	2500	91.55%	91.70%	0.15%
	Han et al. (2020)	VGG-16	IF^**^	2048	93.63%	93.63%	0.01%
	Bu et al. (2022)	VGG-16	IF^**^	32	95.54%	N/A	N/A
	Deng and Gu, 2021)	VGG-16	IF^**^	128	92.24%	92.09%	−0.15%
	Li et al. (2021a)	VGG-16	IF^**^	128	95.65%	95.72%	0.07%
	Hu et al. (2021)	ResNet-110	IF^**^	350	93.02%	93.47%	0.45%
	Li et al. (2021a)	MobileNet	IF^**^	128	91.70%	92.48%	0.78%
	**Ours**	**LeNet-5**	**Ca-LIF** ^**^	**128**	**78.44%**	**78.70%**	**0.26%**
	**Ours**	**VGG-9**	**Ca-LIF** ^**^	**128**	**93.63%**	**93.71%**	**0.08%**
	**Ours**	**ResNet-11H**	**Ca-LIF** ^**^	**128**	**93.58%**	**93.52%**	**-0.06%**
	**Ours**	**MobileNet-20**	**Ca-LIF** ^**^	**128**	**91.71%**	**92.20%**	**0.49%**
	**Ours**	**VGG-16**	**Ca-LIF** ^**^	**128**	**93.68%**	**94.02%**	**0.34%**
CIFAR-100	Kundu et al. (2021)	ResNet-12	LIF^**^	120	63.02%	63.52%	0.50%
	Sengupta et al. (2019)	ResNet-20	IF^*^	2500	64.09%	68.72%	4.63%
	Han et al. (2020)	ResNet-20	IF^**^	2048	67.82%	68.72%	0.9%
	Deng and Gu, 2021)	ResNet-20	IF^**^	128	69.49%	67.08%	−2.41%
	Kundu et al. (2021)	VGG-11	LIF^**^	100	64.98%	67.40%	2.42%
	Sengupta et al. (2019)	VGG-16	IF^*^	2500	70.77%	71.22%	0.45%
	Han et al. (2020)	VGG-16	IF^**^	2048	70.93%	71.22%	0.29%
	Deng and Gu, 2021)	VGG-16	IF^**^	128	70.47%	70.62%	0.15%
	Hu et al. (2021)	ResNet-110	IF^**^	350	70.62%	72.03%	1.45%
	Li et al. (2021a)	MobileNet	IF^**^	128	71.02%	73.23%	2.21%
	**Ours**	**VGG-9**	**Ca-LIF** ^**^	**128**	**65.95%**	**67.48%**	**1.53%**
	**Ours**	**ResNet-11H**	**Ca-LIF** ^**^	**128**	**69.36%**	**69.55%**	**0.19%**
	**Ours**	**MobileNet-20**	**Ca-LIF** ^**^	**128**	**68.20%**	**69.25%**	**1.05%**
	**Ours**	**VGG-16**	**Ca-LIF** ^**^	**128**	**72.85%**	**72.47%**	**-0.38%**
Tiny-ImageNet	Kundu et al. (2021)	VGG-16	LIF^**^	150	52.70%	57.00%	4.3%
	**Ours**	**VGG-9**	**Ca-LIF** ^**^	**128**	**52.80%**	**54.39%**	**1.59%**
	**Ours**	**ResNet-11H**	**Ca-LIF** ^**^	**128**	**53.18%**	**54.26%**	**1.08%**
	**Ours**	**MobileNet-20**	**Ca-LIF** ^**^	**128**	**59.70%**	**60.7%**	**1.00%**

^*^Using hard-reset mechanism, i.e., reset by clearing V_m_ to 0. ^**^Using soft-reset mechanism, i.e., reset by subtracting V_th_ from V_m_ (and by adding V_th_ to V_m_ upon a negative firing, as in our Ca-LIF neuron model). The bold values indicate the performance of our ANN-SNN conversion method. ^#^Number of Time-steps.

For the MNIST dataset, the accuracies of our SNNs are a little higher than full-precision ANNs due to the higher accuracies of the QAT-trained ANNs. When it comes to CIFAR-10, the accuracy of our VGG-9 (93.63% for *T* = 128) surpasses those provided by Diehl et al. (2015), Sengupta et al. (2019), and Kundu et al. (2021). Using fewer time steps, our ResNet-11H on CIFAR-10 (93.58% for *T* =128) exceeds those using the same structure provided by Diehl et al. (2015) and Sengupta et al. (2019) and the deeper ResNet structure provided by Sengupta et al. (2019), Han et al. (2020), Hu et al. (2021), and Deng and Gu (2021). As compared to Bu et al. (2022) (92.35% for *T* = 64), our ResNet-11H (93.44% for *T* = 64) also has a better performance. The reason that the accuracy of our ResNet-11H is lower than that of Deng et al. (2022) will be discussed in section 4.4. The accuracy of our MobileNet-20 is slightly superior to that of Li et al. (2021a), while our VGG-16 on CIFAR-10 is preferable to Sengupta et al. (2019) and Han et al. (2020) in terms of both accuracy and latency (i.e., number of time steps). The accuracy of our VGG-16 is a little lower than that provided by Bu et al. (2022) and Li et al. (2021a) due to their high-accuracy baseline full-precision ANN, while our method relies on the QAT framework which produces a less-accurate ANN model for conversion. Fortunately, our method requires no complex operations like modifying the loss function mentioned by Bu et al. (2022) or post-processing calibrations mentioned by Li et al. (2021a). For the CIFAR-100 dataset, our ResNet-11H SNN also transcends more complex ResNet structures (Sengupta et al., 2019; Han et al., 2020; Hu et al., 2021) while falling behind (Deng and Gu, 2021). The accuracy and the latency metrics of our VGG-16 on CIFAR-100 outperform those using the same network architecture (Sengupta et al., 2019; Han et al., 2020; Deng and Gu, 2021).

Regarding the Tiny-Image-Net dataset, the overall performance (accuracy, latency, and ANN-to-SNN accuracy loss) of all our networks defeat those of Kundu et al. (2021).

In general, Table 2 indicates that our SNNs converted from QAT-trained ANNs can achieve competitively high recognition accuracies across all the used network structures on the benchmark image datasets, when compared to the similar network topologies used in other studies. Our SNN accuracy loss with respect to the corresponding full-precision ANNs also keeps as low as that of other studies. Moreover, in our study, the low-precision data quantization in ANNs allows an intermediate temporal window of *T* = 128 time steps for the converted SNNs to complete inference at an acceptable computational overhead on potential neuromorphic hardware platforms.

Table 3 Further uses the VGG-16 structure and CIFAR-10 dataset to test the accuracies of our converted SNNs with varying time steps and compares them with some recent ANN-to-SNN conversion researches. Our study surpasses Han et al. (2020), Han and Roy (2020) and Ding et al. (2021) totally under all time-step configurations. Our SNN accuracy is still comparably competent when using a relatively short time length of *T* = 32 time steps. However, when the time window is as extremely short as *T* = 16 or 8, our SNN accuracies start to obviously lag the ones obtained by Deng and Gu (2021), Bu et al. (2022), and Li et al. (2022b). Similar conclusions can be drawn from Table 4, where our study is compared with other studies on the SNN accuracies on the more challenging CIFAR-100 dataset. Our SNN accuracies are comparable to the others when *T* is 32 time steps or longer, but obviously lower for *T* = 8 and 16 time steps. We deem this accuracy degradation as the cost of adopting an off-the-shelf QAT ANN training toolkit without dedicated optimizations toward low-latency inference as employed in Deng and Gu (2021), Bu et al. (2022), and Li et al. (2022b). The recently emerged direct SNN training methods can also reach a relatively high accuracy while consuming much fewer time steps < 10 (Guo et al., 2021, 2022a,b,c,d; Deng et al., 2022; Kim et al., 2022; Li et al., 2022a). However, evaluating direct SNN training methods is out of the scope of this article.

**Table 3:** Testing accuracies of the SNNs on CIFAR-10 with different time steps.

**Network structure**	**Ref**.	**Full-precision ANN Acc**.	***T* = 8**	***T* = 16**	***T* = 32**	***T* = 64**	***T* = 128**
**VGG-16**	Han et al. (2020)	93.63%	-	-	60.30%	90.35%	92.41%
	Han and Roy (2020)	93.63%	-	-	-	92.79%	93.27%
	Ding et al. (2021)	92.82%	-	57.90%	85.40%	91.15%	92.51%
	Deng and Gu, 2021)	92.09%	-	92.29%	92.29%	92.22%	92.24%
	Li et al. (2021a)	95.72%	-	-	93.71%	95.14%	95.65%
	Bu et al. (2022)	95.52%	94.95%	95.40%	95.54%	95.55%	95.59%
	Li et al. (2022b)	95.60%	91.41%	93.64%	94.81%	-	-
	**Ours**	**94.02%**	**80.28%**	**90.35%**	**93.10%**	**93.32%**	**93.68%**

The bold values indicate the testing performance of our conversion SNN on CIFAR-10 with different time steps.

**Table 4:** Testing accuracies of the SNNs on CIFAR-100 with different time steps.

**Network structure**	**Ref**.	**Full-precision ANN Acc**.	***T* = 8**	***T* = 16**	***T* = 32**	***T* = 64**	***T* = 128**
**VGG-16**	Han et al. (2020)	71.22%	-	-	-	-	63.76%
	Han and Roy (2020)	71.22%	-	-	-	-	69.86%
	Deng and Gu (2021)	70.49%	-	65.94%	69.80%	70.35%	70.47%
	Li et al. (2021a)	77.89%	-	-	73.55%	76.64%	77.40%
	Bu et al. (2022)	76.28%	73.96%	76.24%	77.01%	77.10%	77.05%
	Li et al. (2022b)	77.93%	64.13%	72.23%	75.53%	-	-
	**Ours**	72.47%	**58.58%**	**66.32%**	**71.71%**	**72.68%**	**72.85%**

The bold values indicate the testing performance of our conversion SNN on CIFAR-10 with different time steps.

The concept of a negative spike has also been proposed by Kim et al. (2020). However, this work differs from theirs mainly in two aspects. First, the neuron model by Kim et al. (2020) has no membrane potential leakage. Rather, it adopts an extra constant input current to represent the bias term in the ANN ReLU. By contrast, our Ca-LIF model naturally incorporates the bias term in the more bio-plausible leakage term. Second and more importantly, the purposes of firing negative spikes are different. The negative spike mentioned by Kim et al. (2020) is only for modeling the negative part of the leaky-ReLU unit widely required in object detection, while our Ca-LIF neuron uses negative spikes to counter-balance the early emitted positive spikes so that when the net input *z*_s_ in Equation (3b) aggregated over the entire time window *T* is negative, the final signed spike count can be zero, which thus closely emulates the quantized ReLU function in classification tasks, as explained in Section 3.1 and 3.2. Some previous ANN-to-SNN works do not adopt such methods but employed more complex threshold/weight balancing operations required to compensate for the early emitted positive spikes (Diehl et al., 2015; Rueckauer et al., 2017; Han et al., 2020; Ho and Chang, 2021; Liu et al., 2022). In this regard, although judging the sign of the spikes puts forward marginally additional computational overhead, it considerably eliminates the tedious post-conversion steps like threshold/weight balancing.

One limitation of the proposed QAT ANN-to-SNN conversion framework, as well as other ANN-to-SNN conversion methods, is that the input spike coding can only employ a rate-coding paradigm, where input spike frequency or count is proportional to the pixel intensity to be encoded. This requires multiple to dozens of spikes for each pixel. These ANN-to-SNN conversion methods cannot accommodate the more computationally efficient temporal coding scheme (Mostafa, 2018), where each pixel is encoded into only one spike whose precise emission time is conversely proportional to the pixel intensity, and each neuron in the SNN is only allowed to fire at most once in response to an input sample. However, as mentioned earlier, since our method can adapt to any available QAT training toolkit, we can resort to those supporting binary or ternary activations, so that the total spikes propagated through our converted SNNs would be largely reduced, with the required inference time window length considerably shortened. Therefore, the gap between the computational overheads of our converted SNNs and the one mentioned by Mostafa (2018) using temporal coding can be well bridged.

## 5. Conclusion

This study proposes a ReLU-equivalent Ca-LIF spiking neuron model and a QAT-based ANN-to-SNN conversion framework requiring no post-conversion operations, to achieve comparably high SNN accuracy in object recognition tasks with an intermediately short temporal window ranging from 32 to 128 time steps. We employed an off-the-shelf PyTorch QAT toolkit to train quantized deep ANNs and directly exported the learned weights to SNNs for inference without post-conversion operations. Experimental results demonstrated our converted SNNs of typical deep network structures can obtain competitive accuracies on various image datasets compared to previous studies while requiring a reasonable number of time steps for the inference. The proposed approach might also be applied to deploy deeper SNN architectures such as MobileNetv2 and VGG-34. Our future research will also include hardware implementation for SNN inference based on our Ca-LIF neurons.

## Data availability statement

Publicly available datasets were analyzed in this study. The MNIST dataset is available at http://yann.lecun.com/exdb/mnist/. The CIFAR-10 and CIFAR-100 is accessible at https://www.cs.toronto.edu/~kriz/cifar.html. The Caltech-101 dataset and the Tiny-ImageNet can be found at https://www.kaggle.com/c/tiny-imagenet.

## Author contributions

HG conceptualized the problem, implemented the algorithm, performed the experiments, and wrote the original manuscript. CS conceptualized the problem, supervised the work, funded the project, and revised the manuscript. MT revised the manuscript and supervised the work. JH, HW, and TW guided the implementation and tested the algorithms. ZZ collected the datasets. JY and YW revised the manuscript. All authors contributed to the article and approved the submitted version.
